# Correction: Synthesis of α,β-unsaturated esters of perfluoropolyalkylethers (PFPAEs) based on hexafluoropropylene oxide units for photopolymerization

**DOI:** 10.1039/d0ra90036b

**Published:** 2020-05-06

**Authors:** Céline Bonneaud, Mélanie Decostanzi, Julia Burgess, Giuseppe Trusiano, Trevor Burgess, Roberta Bongiovanni, Christine Joly-Duhamel, Chadron M. Friesen

**Affiliations:** Trinity Western University, Department of Chemistry 7600 Glover Road Langley British Columbia V2Y 1Y1 Canada chad.friesen@twu.ca; Ingénierie et Architectures Macromoléculaires, Institut Charles Gerhardt, Ecole Nationale Supérieure de Chimie de Montpellier (UMR5253-CNRS) 240 Avenue Prof Emile Jeanbrau 34296 Montpellier Cedex 5 France; Department of Applied Science and Technology, Politecnico di Torino 10128 Torino Italy

## Abstract

Correction for ‘Synthesis of α,β-unsaturated esters of perfluoropolyalkylethers (PFPAEs) based on hexafluoropropylene oxide units for photopolymerization’ by Céline Bonneaud *et al.*, *RSC Adv.*, 2018, **8**, 32664–32671.

The authors regret that a consistent structure error appears in Schemes 4–6 and the graphical abstract of the original article. The definition shown for R_F_ is incorrect, and the correct definition is R_F_ = –CF(CF_3_)(OCF_2_CF(CF_3_))_*n*_OCF_2_CF_2_CF_3_. The correct versions of Schemes 4–6 are shown below, and the graphical abstract has been updated in the online version of the original article.

**Scheme 4:**
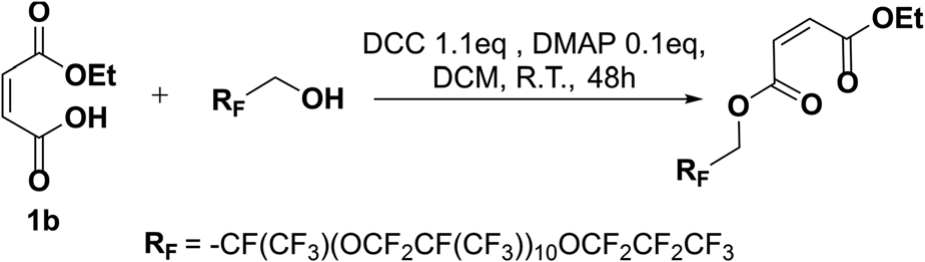
Synthesis scheme of maleate oligo(HFPO) using DCC and DMAP.

**Scheme 5:**
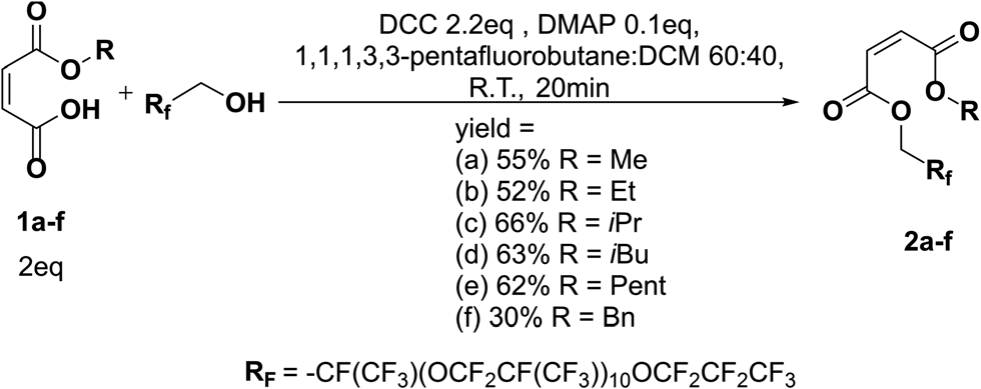
Reaction scheme of Steglich esterification with oligo(HFPO) methylene alcohol *M*_w_ ∼ 2000 g mol^−1^.

**Scheme 6:**
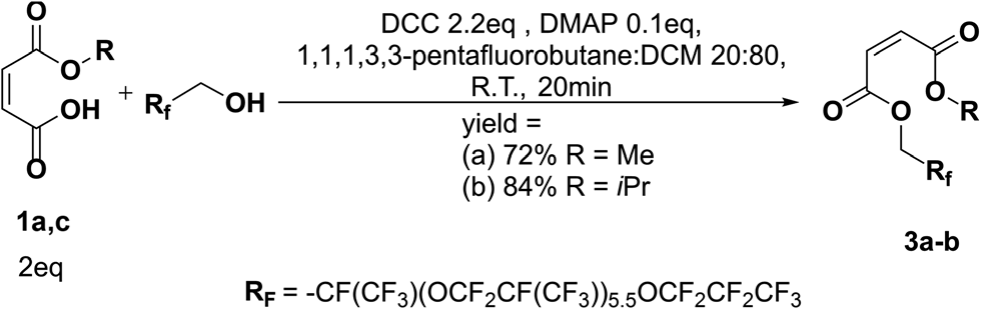
Reaction scheme of Steglich esterification on oligo(HFPO) alcohol *M*_w_ ∼ 1250 g mol^−1^.

The Royal Society of Chemistry apologises for these errors and any consequent inconvenience to authors and readers.

## Supplementary Material

